# Formononetin, a Beer Polyphenol with Catabolic Effects on Chondrocytes

**DOI:** 10.3390/nu15132959

**Published:** 2023-06-29

**Authors:** María Guillán-Fresco, Eloi Franco-Trepat, Ana Alonso-Pérez, Alberto Jorge-Mora, Verónica López-López, Andrés Pazos-Pérez, María Piñeiro-Ramil, Rodolfo Gómez

**Affiliations:** Musculoskeletal Pathology Group, Health Research Institute of Santiago de Compostela (IDIS), Santiago University Clinical Hospital SERGAS, 15706 Santiago de Compostela, Spainveronica.lopez.lopez@sergas.es (V.L.-L.);

**Keywords:** phytoestrogens, osteoarthritis, growth plate

## Abstract

Beer consumption has been identified as a risk factor for osteoarthritis (OA), a rheumatic disease characterised by cartilage degradation, joint inflammation, and eventual joint failure. One of the main isoflavonoids in beer is formononetin (FNT), an estrogenic compound also found in multiple plants and herbs. In this study, we aimed to investigate the effect of FNT on chondrocyte viability, inflammation, and metabolism. Cells were treated with FNT with or without IL-1β for 48 h and during 7 days of differentiation. Cell viability was determined via MTT assay. Nitrite accumulation was determined by Griess reaction. The expression of genes involved in inflammation and metabolism was determined by RT-PCR. The results revealed that a low concentration of FNT had no deleterious effect on cell viability and decreased the expression of inflammation-related genes. However, our results suggest that FNT overexposure negatively impacts on chondrocytes by promoting catabolic responses. Finally, these effects were not mediated by estrogen receptors (ERs) or aryl hydrocarbon receptor (AhR). In conclusion, factors that favour FNT accumulation, such as long exposure times or metabolic disorders, can promote chondrocyte catabolism. These data may partially explain why beer consumption increases the risk of OA.

## 1. Introduction

Osteoarthritis (OA) is the most common rheumatic disease and one of the most disabling pathologies worldwide [1,2,3]. It is clinically characterised by narrowed joint space owing to a progressive cartilage degradation [1,2,3,4]. However, periarticular tissues are also normally compromised, leading to the whole joint failure [1,2,3]. As a result of these disturbances, symptoms such as inflammation, stiffness, loss of function, and joint pain appear, increasing patients’ dependency [1,2,5]. Moreover, OA is correlated to considerable social economic burdens thanks to its high incidence in society [1,2,5]. Although OA aetiology is not fully understood, there are different factors that can contribute to its progression such as certain genetic profiles, ethnicity, age, sex, metabolic diseases, mechanical stress, inflammation, and diet habits [1,2,3,4,5,6,7].

It is well-known that inflammation plays a key role in OA catabolic process and, therefore, in the progression of the pathology [2,4,6,7]. Specifically, cytokines such as interleukin 1 beta (IL-1β), interleukin 6 (IL-6), and tumour necrosis factor alpha (TNF-α) have been strongly related to these responses in patients who suffer from OA [2,6,7]. Inflammatory processes cause oxidative stress through the action of reactive oxygen species. This leads to the damage of chondrocytes, as well as to the degradation of the extracellular matrix (ECM), due in part to its negative impact on aggrecan (ACAN) and collagen type II alpha 1 (COL2A1) synthesis [2,8]. Moreover, inflammation involves the activation of matrix metalloproteinases (MMPs) such as MMP13, contributing in this way to cartilage ECM degradation [6,8].

Diet has been proposed as one of the potentially modifiable risk factors in OA’s development. Specifically, beer consumption has been linked to OA progression [9]. However, its intake has also been related to bone anabolic effects [10,11,12]. Among the active compounds present in beer, flavonoids stand out [13]. Specifically, formononetin (FNT) is one of the major isoflavonoids present in beer. The FNT content in beer ranges from 0.19 to 14.99 nM, which is closely related to its area of origin. Consequently, the FNT concentration is different among beer brands [13]. Nonetheless, FNT is not exclusively from beer. This active compound, to which estrogenic and osteoinductive properties have been attributed [11,14,15], is also found in other beverages such as coffee [16,17,18] or tea [18], and in a wide number of plants and herbs like red clover or leguminous plants [11,12,13,15]. Thus, FNT is widely present in diet. However, insufficient research is currently available to assess the average of FNT consumption worldwide. Nonetheless, it is known that Asian populations are more exposed to phytoestrogens than Western or Eastern populations because of their isoflavonoid-rich diet, based on the consumption of soybeans and legumes [19,20].

Several studies have suggested the impact of isoflavonoids’ consumption on health [21,22,23,24]. Nevertheless, the effect of FNT on chondrocytes has not yet been extensively investigated [25,26,27,28]. Besides a potential effect on articular chondrocytes, this phytoestrogen could also impact the growth plate chondrocytes. Currently, there is an emerging concern about the impact of isoflavonoids in infants owing to their potential overexposure during prenatal and neonatal periods [29]. Isoflavonoids can be found in breast milk [30,31]; amniotic fluid [32]; and infant food and formulas, specially soy-based formulas [29,30,33,34]. Importantly, phytoestrogens’ safety index is lower in children than in adults [33]. Even so, the potential impact of FNT on growth plate chondrocytes remains uninvestigated to date.

Because of its structural similarity to mammalian estrogen and its ability to bind to estrogen receptors (ERs), such as ER alpha (ERα) and beta (ERβ), it has been suggested that FNT could exert its actions through binding to these receptors [12,14,15,35,36,37]. Related to this, it has been demonstrated that the aryl hydrocarbon receptor (AhR), a functional receptor present in articular and growth plate cartilages [38,39], can establish crosstalk with ERs’ signalling pathways, hindering their ability to bind to estrogen response elements [40,41]. Accordingly, it is well-known that FNT is a potent agonist of human AhR [41]. However, nothing is known about the effects of this isoflavonoid in chondrocyte catabolic and inflammatory responses.

For this reason, in this in vitro study, we focus on the role of FNT in inflammatory and catabolic chondrocyte responses. Moreover, we tried to elucidate whether FNT actions in chondrocytes are due to its capacity to modulate estrogenic pathways. The results obtained in this study showed that FNT does not alter chondrocyte viability and has moderate anti-inflammatory activity at the lowest concentration tested. However, FNT promotes chondrocyte catabolic responses by itself and in combination with the inflammatory stimuli in an estrogen-independent way.

## 2. Materials and Methods

### 2.1. Reagents

ATDC5 murine chondrogenic cell line was obtained from RIKEN Cell Bank (Koyadai, Tsukuba, Ibaraki, Japan). Dulbecco’s Modified Eagle’s Medium (DMEM) supplemented with Ham’s F-12, Fetal Bovine Serum (FBS), antibiotics Penicillin-Streptomycin, Transferrin, Sodium Selenite, L-Glutamine, Trypsin, TRI Reagent, FNT, 3′methoxy-4′nitroflavone (3-MF), mouse IL-1β, MTT reactive, chloric acid, SDS, and Griess reactives (phosphoric acid, sulphanilamide, and alpha-naftil-etil-enamide) were purchased from Sigma Aldrich (St. Louis, MO, USA). ZK164015 was acquired from Cienytech (Santiago de Compostela, Spain). Insulin (Actrapid 100 UI/ML) was kindly donated by the Pharmacy Service of the Clinical Hospital of Santiago de Compostela. E.Z.N.A. Total RNA Kit I was bought from Omega BIO-TEK (Norcross, GA, USA). Digestion kit was purchased from Lucigen (Middelton, WI, USA). High-capacity cDNA Reverse Transcription Kit was bought from Applied Biosystems, Life Technologies (Grand Island, NY, USA). MasterMix PCR was purchased from ThermoFisher Scientific (Waltham, MA, USA). IL-6, neutrophil gelatinase-associated lipocalin (LCN2), C-C motif chemokine 2 (CCL2), COL2A1, ACAN, type X collagen (COLX), SRY-Box transcription factor 9 (SOX9), and MMP13 primers were purchased from Sigma Aldrich. Cell culture plates were purchased from ThermoFisher Scientific.

### 2.2. Experimental Design

#### 2.2.1. Forty-Eight-Hour Experiments

ATDC5 cells were seeded on six-well plates (25 × 10^4^ cells/well) in DMEM/Ham’s F-12 medium supplemented with 5% FBS, penicilin-streptomicyn (2%), L-glutamine (2%), transferrin (10 μg/mL), and sodium selenite (0.03 nM) (basal medium). Once cells adhered to the plates, they were serum-deprived. Then, 24 h after seeding, an FBS-supplemented medium was added, and cells were treated with 12.5 or 25 μM FNT in the presence or absence of IL-1β. Then, 48 h after FNT treatment, cells were lysed, RNA was obtained, and RT-PCR was performed.

#### 2.2.2. Cell Differentiation

Cell differentiation was performed for 7 days. Cells were seeded in 100 mm culture plates (5 × 10^5^ cells/plate for the day 0 plate and 3 × 10^4^ cells/plate for day 7 plate). Once they adhered to the plate, the medium was changed for differentiation medium, which consisted of basal medium with insulin (10 μg/mL), to promote cell differentiation [42,43,44,45]. Then, the differentiation medium was replaced three times per week (FNT and/or IL-1β were added in every replacement) and cells were lysed after 7 days of differentiation. Afterwards, RNA was extracted and RT-PCR was performed.

### 2.3. Cell Treatments

ATDC5 cells were treated with IL-1β (0.1 ng/mL); FNT 5, 12.5, and 25 μM; ZK164015 (ZK) 0.1 μM; and 3′methoxy-4′nitroflavone (3-MF) 10 μM. IL-1β was diluted in water, whereas FNT, ZK, and 3-MF stocks were dissolved in DMSO, following the manufacturer’s instructions.

### 2.4. Cell Viability Assay

Cell viability was examined using a colorimetric MTT assay, as previously described [46]. Briefly, 8 × 10^3^ cells were seeded on 96-well plates and treated with FNT 5, 12.5, and 25 μM in presence or absence of IL-1β (0.1 ng/mL). After 48 h of treatment, cells were incubated with MTT 5 mg/mL over 4 h at 37 °C. Then, 100 μL of solubilizing agent was added per well and cells were incubated overnight. After formazan salt was dissolved, absorbance was measured at 550 nm with one NanoDrop One^®^ spectrophotometer (Thermo Fisher Scientific).

### 2.5. Nitrite Assay

ATCD5 cells were seeded in six-well plates (25 × 10^4^ cells/well). After cell adhesion to the plate, they were serum-deprived and treated with FNT 25 μM in the presence or absence of IL-1β, as aforementioned. After 24–48 h of treatment, nitrite accumulation in the culture medium was determined using the Griess reaction, as previously described [46].

### 2.6. RNA Extraction and Quantitative Real-Time PCR

Once the experiments were concluded, cells were lysed and RNA was extracted using E.Z.N.A Total RNA Kit I according to the manufacturer’s protocol. Afterwards, RNA was purified using the Lucigen digestion kit following the supplier’s instructions. For the reverse transcription, the High-capacity cDNA Reverse Transcription Kit was used. The expression of IL-6, LCN2, CCL2, COL2A1, ACAN, SOX9, MMP13, and COLX was measured by RT-PCR using the MasterMix PCR (ThermoFisher Scientific) in combination with specific primers in a QuantStudio 3 (ThermoFisher) (Table 1). Relative quantitation was performed using the ΔΔCt Comparative Method. The results were expressed as fold change versus unstimulated control.

### 2.7. Docking Analysis

ERα, ERβ, and AhR structures were obtained from the RCSB Protein Data Bank (PDB) [47] and optimized using PyMol software 2.5.2 [48]. To complement the receptor structures, we accessed the PubChem library to acquire the structures of agonists and drugs. EasyDockVina 2.2 software was used to optimize structures and to perform the molecular docking analysis. The results are shown in Gibbs free energy units (Kcal/mol) and ranked from lowest (strongest interaction) to highest (weakest interaction) for all possible conformations.

### 2.8. Statistical Analysis

Data were reported as mean ± standard error of the mean (SEM) for at least three independent experiments. Statistical analysis was performed by analysis of variance followed by the Tukey’s multiple comparisons test or *t*-test analysis using the Prism computerized package (GraphPad Prism 8 Software Inc., La Jolla, CA, USA). A difference was considered significant when *p* < 0.05.

## 3. Results

### 3.1. FNT Does Not Alter Chondrocyte Viability

**Figure 1:**
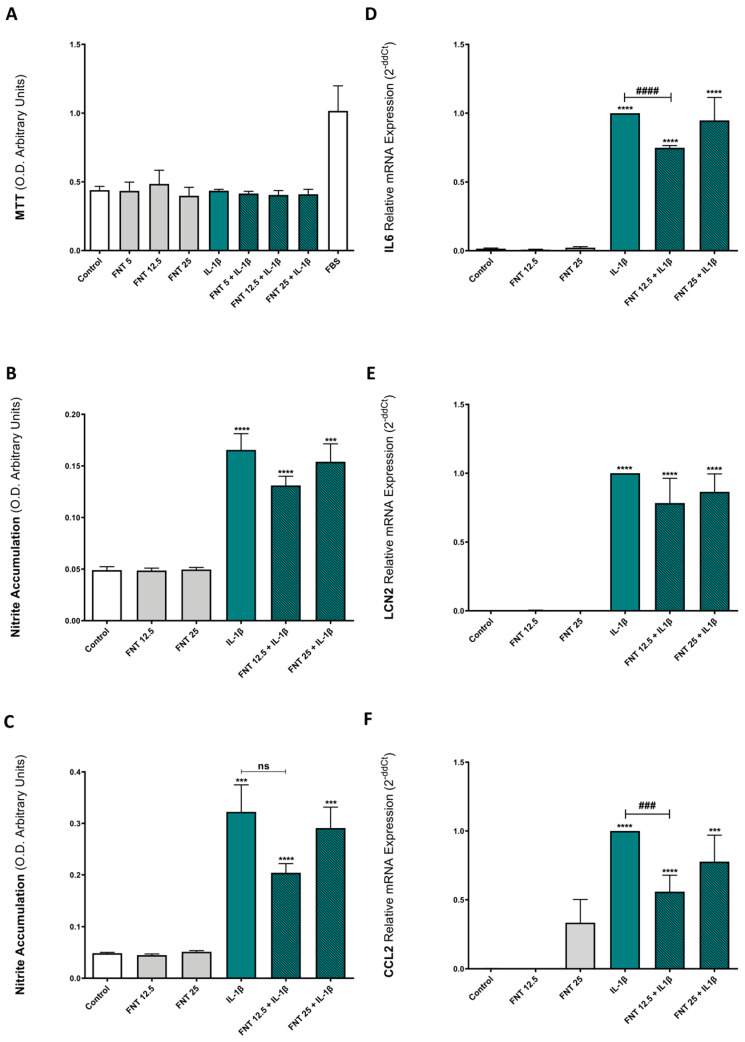
FNT effect on ATDC5 cells’ viability and inflammation. (**A**) ATDC5 cells were incubated for 48 h with FNT (5, 12.5, and 25 μM), IL-1β (0.1 ng/mL), and the combination of both compounds to test their effect on cell viability by an MTT assay. The results are expressed in arbitrary optical density (O.D.) units. Experiments were performed at least in triplicate with eight independent observations for each experiment and treatment. (**B**,**C**) ATDC5 cells were stimulated for 24 (**B**) and 48 (**C**) hours with FNT 12.5 or 25 μM in the presence or absence of IL-1β (0.1 ng/mL). NO production was evaluated as nitrite accumulation in the culture medium using the Griess reaction. The results are expressed in arbitrary O.D. units. (**D**–**F**) IL-6, LCN2, and CCL2 mRNA expression were determined by RT-PCR after 48 h of stimulation with FNT 12.5 or 25 μM in the presence or absence of IL-1β (0.1 ng/mL) on ATDC5 cells. The results are expressed as mean ± SEM of three independent experiments. *** *p* < 0.001 and **** *p* < 0.0001 statistics vs. unstimulated control; ^###^ *p* < 0.001 and ^####^ *p* < 0.0001 statistics vs. stimulated control. ns: non significant.

Beer has been associated with anabolic actions in bone tissue [10,11,12], as well as with OA development [9]. Thus, we explored the effects of FNT, one of the main isoflavonoids present in beer [13], on the metabolic activity of chondrocytes by means of an MTT assay. The data obtained revealed that 48 h FNT stimulation did not alter ATDC5 cells’ metabolic activity/viability (Figure 1A). In fact, it did not have any effect on cell viability, even in the presence of IL-1β, a pro-inflammatory cytokine typically associated with OA inflammatory and catabolic environment [2,6,7].

### 3.2. FNT Has Moderate Anti-Inflammatory Activity Only at Low Concentrations

Considering the key role of a pro-inflammatory environment on OA progression [2,6,7], we aimed to investigate the effect of FNT on chondrocyte’s inflammatory responses. To induce an inflammatory response in ATDC5 chondrocytes, we stimulated them with IL-1β over 24 h and 48 h. Inflammatory responses were evaluated by the determination of nitric oxide (NO) production (evaluated as nitrite accumulation), as well as by the determination of the gene expression (mRNA) of the inflammatory markers IL-6, LCN2, and CCL2.

As shown in Figure 1B,C, FNT did not reduce NO production induced by IL-1β in the 24 or 48 h stimulation. Likewise, 25 μM FNT did not exhibit a significant effect on IL-6-, LCN2-, and CCL2-induced gene expression (Figure 1D–F), although 12.5 μM FNT moderately, but significantly, decreased IL-6 (Figure 1D) and CCL2 (Figure 1F) induction by IL-1β.

### 3.3. FNT Promotes Chondrocyte Catabolic Responses

OA is primarily characterised by progressive cartilage degradation, where chondrocyte’s anabolic responses are inhibited and catabolic ones are promoted [49,50,51,52,53]. Accordingly, we evaluated the effect of FNT on the anabolic and catabolic chondrocyte responses.

With this aim, ATDC5 cells were cultured over 48 h in the presence or absence of FNT and/or IL-1β. To test the anabolic/catabolic effects of FNT, we measured the expression of some relevant genes that play a key role in cartilage maintenance: COL2A1 and ACAN, both indispensable for ECM integrity [54,55]; SOX9, a transcription factor related to the chondrocyte phenotype preservation [56,57]; and MMP13, a metalloproteinase involved in cartilage degradation [49,50,51].

**Figure 2:**
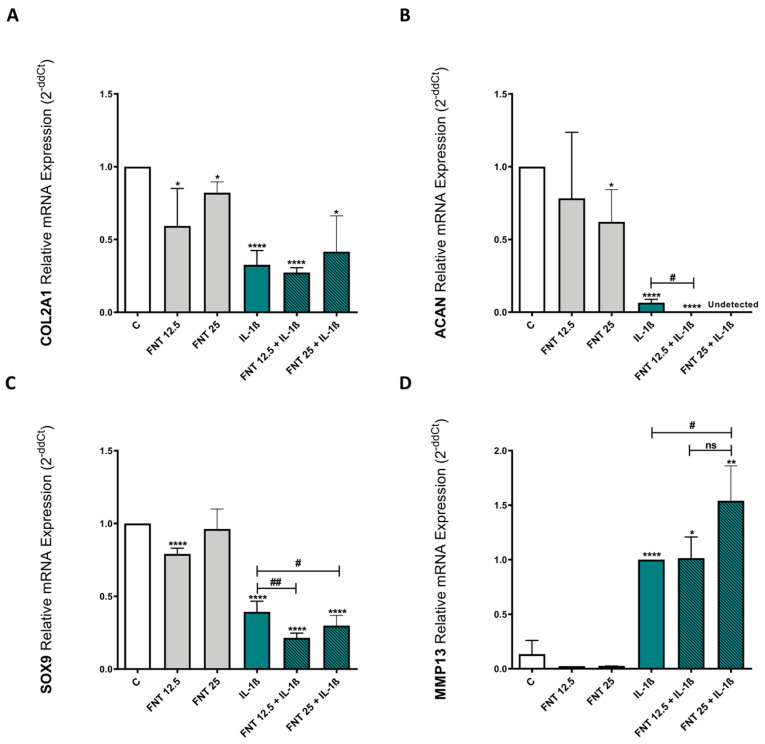
FNT effect on ATDC5 anabolic and catabolic responses. (**A**–**D**) ATDC5 cells were stimulated over 48 h with FNT 12.5 or 25 μM in the presence or absence of IL-1β (0.1 ng/mL). COL2A1, ACAN, SOX9, and MMP13 mRNA expressions were determined by RT-PCR after 48 h of stimulation. The results are expressed as mean ± SEM of three independent experiments. * *p* < 0.05; ** *p* < 0.01 and **** *p* < 0.0001 statistics vs. unstimulated control. ^#^ *p* < 0.05 and ^##^ *p* < 0.01 statistics vs. stimulated control. ns: non significant.

The obtained results showed that 48 h of stimulation with 25 μM FNT discretely reduced the expression of COL2A1 and ACAN on ATDC5 cells in a significant way. Treatment with 12.5 μM FNT also reduced the expression of COL2A but not ACAN (Figure 2A,B). In addition, 12.5 μM FNT reduced the expression of SOX9 (Figure 2C). Moreover, these results revealed that FNT has a deleterious impact on ATDC5 cells when combined with the inflammatory stimuliIL-1β. As shown in Figure 2B,C, FNT enhanced IL-1β-mediated reduction of the expression of ACAN and SOX9, as well as the increase in MMP13 expression promoted by IL-1β stimulation (Figure 2D).

### 3.4. FNT Negatively Impacts Chondrocyte Differentiation

Considering the effects of FNT on chondrocytes’ anabolism and catabolism and the possibility of infant exposure to this isoflavonoid through breast milk or infant formulas [29,30,31,33,34], we investigated whether FNT could exert an impact on chondrocyte differentiation. Chondrocyte differentiation plays a key role in longitudinal bone growth and is also an essential process for bone fracture healing [58,59,60].

**Figure 3:**
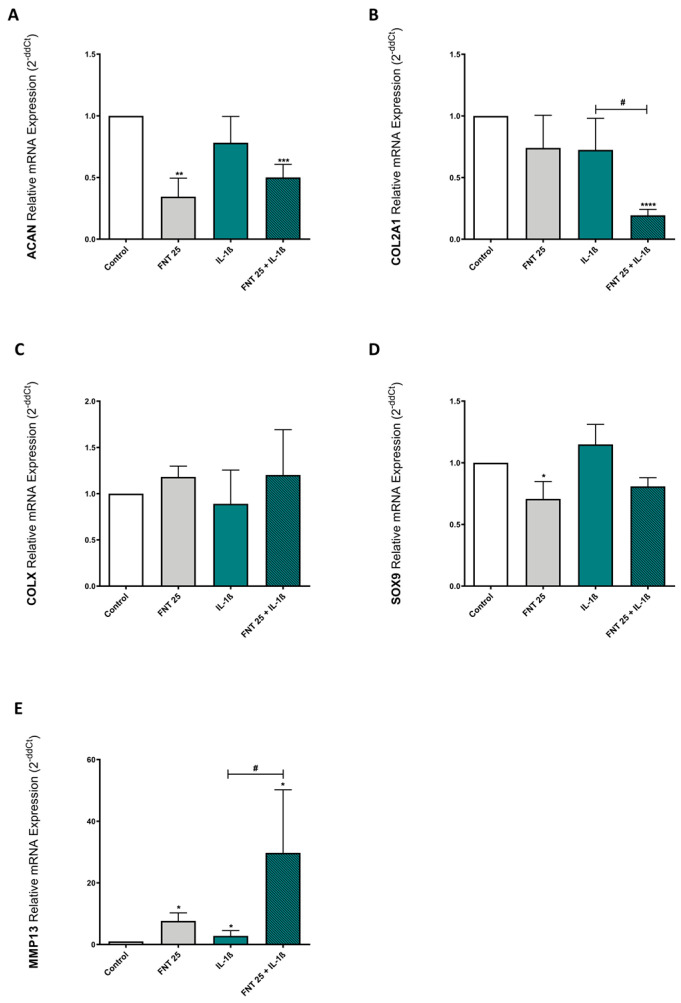
FNT effect on ATDC5 cell differentiation. ATDC5 cells were differentiated over 7 days with FNT 25 μM in the presence or absence of IL-1β (0.1 ng/mL). ACAN (**A**), COL2A1 (**B**), COLX (**C**) SOX9 (**D**), and MMP13 (**E**) mRNA expressions were determined by RT-PCR after 7 days of differentiation. The results are expressed as mean ± SEM of three independent experiments. * *p* < 0.05, ** *p* < 0.01, *** *p* < 0.001, and **** *p* < 0.0001 statistics vs. unstimulated control. ^#^ *p* < 0.05 statistics vs. stimulated control.

To evaluate this, we carried out the differentiation of ATDC5 cells over 7 days in the presence or absence of 25 μM FNT (the concentration with the strongest catabolic effect) and/or IL-1β, and measured the expression of differentiation and catabolic markers by RT-PCR. Our results indicated that FNT significantly diminished ACAN (Figure 3A) but not COL2A1 expression (Figure 3B). However, it also reduced SOX9 expression (Figure 3D) and increased MMP13 expression after 7 days of differentiation, in comparison with the untreated control (Figure 3E). In addition, FNT treatment significantly reduced the expression of COL2A1 and enhanced the expression of MMP13 when combined with the inflammatory cytokine IL-1β (Figure 3B,E). Interestingly, and in contrast with the effect of FNT, continuous stimulation of chondrocytes for 7 days with IL-1β did not induce any significant change in the expression of the studied genes (Figure 3). These results showed that FNT in combination with inflammatory stimuli has a negative impact on chondrocyte differentiation.

### 3.5. FNT Has a Predicted Strong Binding Affinity to ERs and AhR

**Figure 4:**
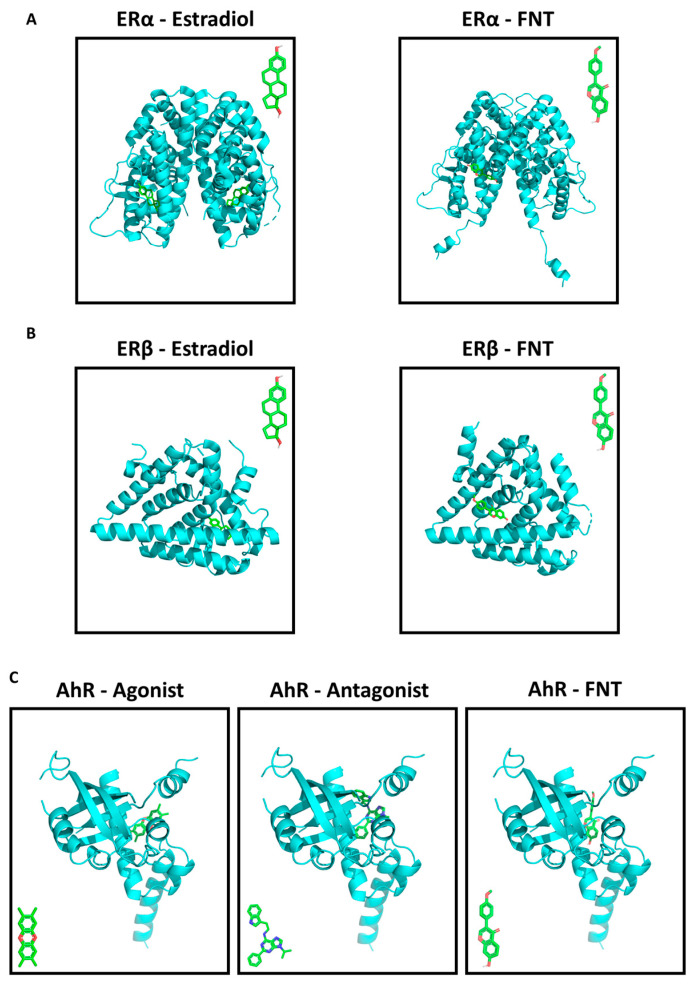
FNT interaction with estrogen receptors ERα and ERβ, as well as AhR. (**A**,**B**) Molecular docking showed that FNT strongly bound to ERα and ERβ, in a different region to their agonist estradiol. (**C**) FNT had a high affinity to bind to AhR, in the same region as its agonist and antagonist. Estradiol, FNT, and AhR agonist and antagonist structures employed for each docking analysis are shown within each panel.

It has been described that FNT could interact with estrogenic receptors ERα and ERβ [12,14,15,35,36], as well as with AhR [40,41], a receptor expressed by growth plate chondrocytes that can establish crosstalk with the ERs [38,39]. Accordingly, we explored the potential interaction of FNT and these receptors by means of a computational approach. FNT showed a strong binding affinity to ERα (−6.8 kcal/mol) and ERβ (−6.5 kcal/mol), but it was not predicted to bind to the same regions as estradiol (Figure 4A,B). The binding affinity of FNT to AhR was similar (−7.7 kcal/mol) and the predicted binding site was in the same region as those of AhR’s agonist and antagonists (Figure 4C). Gibbs free energy values for the nine strongest interactions for each receptor/ligand pair are shown in Supplementary Table S1.

### 3.6. FNT Effects on Chondrocyte Differentiation Are Not Mediated by ERs or AhR

**Figure 5:**
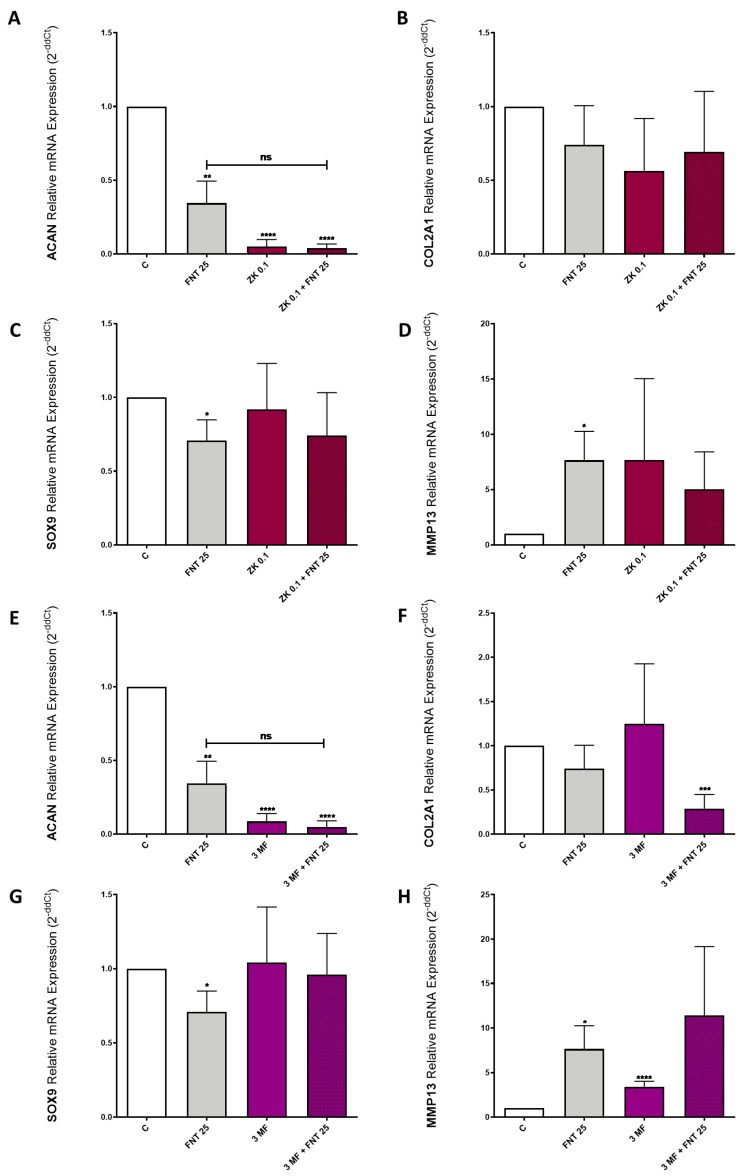
ZK and 3-MF effects on ATDC5 cell differentiation. ATDC5 cells were differentiated over 7 days with FNT 25 μM in the presence or absence of ZK 0.1 μM (**A**–**D**) and 3-MF 10 Μm (**E**–**H**). COL2A1, ACAN, SOX9, and MMP13 mRNA expressions were determined by RT-PCR after 7 days of differentiation. The results are expressed as mean ± SEM of three independent experiments. * *p* < 0.05, ** *p* < 0.01, *** *p* < 0.001, and **** *p* < 0.0001 statistics vs. unstimulated control. ns: non significant.

As an aim of the molecular docking results, we carried out the differentiation of ATDC5 cells over 7 days in the presence or absence of 25 μM FNT; 0.1 μM ZK, an ERα and ERβ receptor antagonist; and 10 μM 3-MF, an AhR receptor antagonist. The obtained data showed that the effect of FNT was not significantly modified by co-treatment with either ZK (Figure 5A–D) or 3-MF (Figure 5E–H). However, the levels of ACAN were reduced in the presence of ZK (Figure 5A) and 3-MF (Figure 5E) alone and in combination with FNT. Besides, 3-MF blockade increased the effects of FNT on the expression of COL2A1 in a non-significant way.

## 4. Discussion

In this study, we investigated the effect of FNT, one of the main isoflavonoids from beer, on chondrocyte inflammation and catabolism. We found that FNT does not affect chondrocyte viability and lowers the expression of inflammation-related genes at a low concentration (12.5 µM). However, at a high concentration (25 µM), it promotes chondrocyte catabolism independently of estrogenic and aryl hydrocarbon receptors. We aimed to investigate the effect of FNT in this context because beer consumption has been identified as a risk factor for OA [9], but the mechanism by which beer consumption may exert its effect has not been described to date. Regarding this matter, Muthuri et al. compared the intake of two alcoholic beverages, beer and wine, highlighting the catabolic effect of beer but not wine on OA [9]. These data suggest the presence of substances in beer other than ethanol that could be responsible for promoting OA. Furthermore, infants and children can also be exposed to FNT and isoflavonoids through breast milk [30,31] and infant food and formulas [29,30,33,34]. Isoflavonoids can exert many biological activities, supposedly because of their structural similarity to estrogens [61]. The FNT effect on bone anabolism has been extensively investigated [11,62], but only a few studies have focused on its effect in cartilage [26,27]. Furthermore, FNT may have opposite effects depending on concentration and pathological status [14,35,63].

Our study shows that FNT has no effect on cell viability either alone or combined with IL-1β at the range of concentrations (5–25 μM) and time (48 h) tested. Other authors also found that 24 h FNT treatment did not affect chondrocyte viability, even at concentrations as high as 100 μM [26,28]. However, it is known that isoflavonoids may inhibit proliferation when used at high concentrations [14,35], and other authors have described that high concentrations of FNT (25–100 μM) can induce apoptosis [64,65].

Some isoflavonoids are known to have anti-inflammatory activity [66]. In our experimental conditions, 12.5 µM FNT reduced the expression of IL-6 and CCL-2 induced by IL1β, but 25 μM FNT had no influence on chondrocyte inflammatory response either alone or in combination with IL-1β. On the contrary, both 12.5 and 25 µM FNT had an effect on the expression of ECM-related genes. FNT treatment promoted a catabolic shift on chondrocytes, as evidenced by the reduction in the expression of COL2A1, ACAN, and SOX9 and, especially, by the induction of the expression of the matrix-degrading enzyme MMP13. In the context of OA, this down-regulation of the expression of anabolic genes together with an up-regulation of the matrix-degrading enzymes would imply a faster progression of the disease. In the context of the growth plate, the reduction in COL2A1 and ACAN will promote chondrocyte hypertrophy [67,68,69], while MMP13 induction will also provoke a faster progression of chondrocyte maturation [70], and thus an early closure of the growth plate. Surprisingly, our results are in contrast with those reported by Cho et al., who described that FNT prevented the catabolic effects of IL-1β, including the promotion of MMP13 expression, in rat chondrocytes [26]. Nonetheless, other authors have reported the ability of FNT to induce the expression of MMP2 and MMP9 through the ERα-enhanced ROCK pathway [37]. Accordingly, it has also been shown that ERα activation can lead to the overexpression of MMP9 and MMP13 [71]. Related to this, estradiol-induced activation of ERα contributes to the loss of collagen and glycosaminoglycans from the cartilage ECM [71].

Interestingly, it has been described that isoflavonoids may stimulate cell growth at low concentrations, but inhibit proliferation at higher concentrations [14,35]. FNT has a biphasic effect on subchondral bone osteoblasts depending on whether they are derived from OA patients or healthy donors, having a pro-anabolic effect on healthy osteoblasts but inhibiting the expression of bone-related markers in OA osteoblasts [63]. Therefore, FNT, as well as other isoflavonoids, may exert antithetical actions depending on both its concentration and the characteristics of the target cells. This might explain why the effects we observed on ATDC5 cells, a well-known in vitro model for studying cartilage ECM biosynthesis [72], are different from those described on rat chondrocytes [26,27].

As a further matter, it has been suggested that FNT can exert its actions through binding to ERs [12,14,15,35,36] and/or AhR [41]. For this reason, we tried to elucidate whether FNT actions in chondrocytes were related to its capacity to modulate estrogenic pathways. In our model, ERs’ blockade did not modulate the effects of FNT on chondrocytes. Estrogen-mimicking molecules, such as isoflavonoids, have been reported to enhance the positive effect of insulin on proteoglycans’ synthesis, supposedly through binding ERs [73]. Nonetheless, other authors have shown that estrogen stimuli induced by phytoestrogens do not significantly affect chondrogenic differentiation of ATDC5 cells [74], which further supports our observation that the FNT effect on ATDC5 cells is ER-independent.

It is known that AhR activation can impact chondrocyte differentiation. In addition, AhR expression positively correlates with chondrocyte maturation during endochondral ossification [38]. Despite the fact that FNT is known to be an agonist of AhR [41], AhR blockade did not modify the effect of FNT on chondrocytes. However, the levels of ACAN were reduced by the blockade of both ERs and AhR, even in the absence of FNT, indicating the impact of the activation of these pathways in the context of cartilage homeostasis. Besides its potential interaction with ERs and AhR, FNT can increase reactive oxygen species’ generation [75], potentially leading to cartilage ECM destruction through other pathways [2,8]. Even so, our results indicate that FNT does not influence the levels of NO production induced by IL-1β, which might suggest the involvement of a different molecular mechanism, independent of ERs, AhR, and oxidative stress, and highly dependent on the cell type and condition.

In conclusion, even though FNT can be beneficial for cartilage homeostasis, exposure to FNT at certain concentrations or physiological/pathological status may promote chondrocyte catabolism. Factors that favour FNT accumulation, such as long times of exposure (e.g., beer consumption throughout life) or metabolic disorders, can lead to this catabolic effect, which is independent of both ERs and AhR. These data may provide a partial explanation for why high beer consumption increases the risk of OA [9].

## Figures and Tables

**Table 1:** List of gene forward primers and reverse primers used in RT-PCR.

Gene	Forward Primer	Reverse Primer
	Sequence (5′-3′)	Sequence (5′-3′)
IL-6	AAGAAATGATGGATGCTACC	GAGTTTCTGTATCTCTCTGAAG
LCN2	ATATGCACAGGTATCCTCAG	GAAACGTTCCTTCAGTTCAG
CCL2	CAAGATGATCCCAATGAGTAG	TTGGTGACAAAAACTACAGC
COL2A1	GCGATGACATTATCTGTGAAG	TATCTCTGATATCTCCAGGTTC
ACAN	CTACCTTGGAGATCCAGAAC	TGGAACACAATACCTTTCAC
SOX9	CTCATTACCATTTTGAGGGG	AAAATACTCTGGTTGCAAGG
MMP13	CTTTAGAGGGAGAAAATTCTGG	CATCATCATAACTCCACACG
COLX	TCATGGGATGTTTTATGCTG	TCTTACTGGAATCCCTTTACTC

## Data Availability

The data used to support the findings of this study are contained within the article. Raw data are available from the corresponding author upon request.

## Supplementary Materials

The following supporting information can be downloaded at https://www.mdpi.com/article/10.3390/nu15132959/s1, Table S1: Gibbs free energy (kcal/mol) values from docking analysis for the nine strongest interactions receptor/ligand.
