# Supplementary material for: Formononetin, a Beer Polyphenol with Catabolic Effects on Chondrocytes
Source: Nutrients. 2023 Jun 29;15(13):2959. doi: 10.3390/nu15132959 (PMC10346251; doi:10.3390/nu15132959)
Supplement: Supplementary file 1 [file nutrients-15-02959-s001.zip › Table S1.pdf]

Table S1. Gibbs free energy (kcal/mol) values from docking analysis for the nine strongest interactions receptor/ligand.

Receptor	ERα		ERβ		AhR		
**Rank**	**Estradiol**	**FNT**	**Estradiol**	**FNT**	**Agonist**	**Antagonist**	**FNT**
1	6.3	6.8	7.0	6.5	6.5	8.0	7.7
2	6.0	6.6	7.0	6.2	6.5	7.9	7.5
3	5.9	6.5	7.0	6.2	6.4	7.7	6.7
4	5.8	6.4	7.0	5.8	6.4	7.5	6.7
5	5.8	6.4	6.9	5.8	6.1	7.5	6.6
6	5.6	6.4	6.6	5.8	6.1	7.2	6.6
7	5.5	6.2	6.5	5.7	6.0	7.1	6.5
8	5.5	6.1	6.5	5.6	6.0	6.9	6.5
9	5.5	6.1	6.4	5.6	5.9	6.3	6.3
